# Neuronal Mitochondrial Calcium Uniporter (MCU) Deficiency Is Neuroprotective in Hyperexcitability by Modulation of Metabolic Pathways and ROS Balance

**DOI:** 10.1007/s12035-024-04148-x

**Published:** 2024-04-23

**Authors:** Laura Bierhansl, Lukas Gola, Venu Narayanan, Andre Dik, Sven G. Meuth, Heinz Wiendl, Stjepana Kovac

**Affiliations:** 1 Department of Neurology With Institute of Translational Neurology, University Hospital Münster, Münster, Germany; 2 Department of Neurology, Medical Faculty and University Hospital, Heinrich Heine University Düsseldorf, Düsseldorf, Germany

**Keywords:** Mitochondrial calcium uniporter (MCU), Hyperexcitability, Neuroprotection, Metabolism, Oxidative stress

## Abstract

Epilepsy is one of the most common neurological disorders in the world. Common epileptic drugs generally affect ion channels or neurotransmitters and prevent the emergence of seizures. However, up to a third of the patients suffer from drug-resistant epilepsy, and there is an urgent need to develop new therapeutic strategies that go beyond acute antiepileptic (antiseizure) therapies towards therapeutics that also might have effects on chronic epilepsy comorbidities such as cognitive decline and depression. The mitochondrial calcium uniporter (MCU) mediates rapid mitochondrial Ca^2+^ transport through the inner mitochondrial membrane. Ca^2+^ influx is essential for mitochondrial functions, but longer elevations of intracellular Ca^2+^ levels are closely associated with seizure-induced neuronal damage, which are underlying mechanisms of cognitive decline and depression. Using neuronal-specific MCU knockout mice (MCU^−/−ΔN^), we demonstrate that neuronal MCU deficiency reduced hippocampal excitability in vivo. Furthermore, in vitro analyses of hippocampal glioneuronal cells reveal no change in total Ca^2+^ levels but differences in intracellular Ca^2+^ handling. MCU^−/−ΔN^ reduces ROS production, declines metabolic fluxes, and consequently prevents glioneuronal cell death. This effect was also observed under pathological conditions, such as the low magnesium culture model of seizure-like activity or excitotoxic glutamate stimulation, whereby MCU^−/−ΔN^ reduces ROS levels and suppresses Ca^2+^ overload seen in WT cells. This study highlights the importance of MCU at the interface of Ca^2+^ handling and metabolism as a mediator of stress-related mitochondrial dysfunction, which indicates the modulation of MCU as a potential target for future antiepileptogenic therapy.

**Supplementary Information:**

The online version contains supplementary material available at 10.1007/s12035-024-04148-x.

## Introduction

Epilepsy is characterized by a heterogeneous spectrum of disorders that have episodic attacks of neuronal hyperexcitability in common. Currently, there are over 25 different antiepileptic drugs (AED) available, which modulate frequency and severity of seizures by affecting ion channels or neurotransmitters. However, up to a third of the patients suffer from refractory epilepsy, and the impact of epilepsy goes well beyond seizures [1].

Epileptogenesis defines a process of converting a non-epileptic brain into one capable of generating spontaneous, recurrent seizures [2]. However, over time, the epileptic neuronal network can expand, and severity of epilepsy might exacerbate. Furthermore, epilepsy is associated with severe comorbidities such as cognitive impairment and affective disorders as depression [3, 4]. Therefore, new therapeutic opportunities are needed to go beyond antiepileptic (antiseizure) therapies towards therapeutics that target the underlying processes leading to the development and progression of the disease and reduction of comorbidities.

In recent years, calcium (Ca^2+^) signaling has become a focus of research and seems to be a promising new target in the development of new antiepileptic drugs [5]. For instance, levetiracetam might exhibit its function at least partially via Ca^2+^ signaling, which leads to the hypothesis of Ca^2+^-mediated epileptogenesis, neuronal plasticity, and neurotoxicity [6].

Neuronal mitochondria have critical cellular functions and are involved in neuronal Ca^2+^ homeostasis. Mitochondrial Ca^2+^ overload, as seen in excitotoxic insults, can lead to the inhibition of ATP synthesis and the induction of the mitochondrial permeability transition pore (mPTP), resulting in mitochondrial dysfunction, cell damage, and neuronal death [7]. Therefore, mitochondrial Ca^2+^ overload could at least in part be involved in the development of acquired epilepsies, and high Ca^2+^ concentrations might also be initiated by seizures and, therefore, could act as an exacerbating factor in epileptogenesis [8].

In 2004, Clapham et al. discovered the existence of a Ca^2+^-selective mitochondrial ion channel. The analysis revealed that the current was mediated by the mitochondrial calcium uniporter (MCU), which controls the stress-dependent transport of Ca^2+^ through the inner mitochondrial membrane [9, 10].

Pharmacological inhibition or genetic ablation of MCU blocks the acute mitochondrial Ca^2+^ influx. It reduces cell death in numerous in vitro models in stress conditions, presumably due to a lower influx of Ca^2+^ and a decreased mPTP opening [9, 11–13]. Furthermore, the MCU inhibitor (Ru360) attenuates neuronal death in rat hippocampal neurons after a pilocarpine-induced status epilepticus [14], and mice with global MCU deletion showed a disturbance in the generation of gamma oscillation and sharp-wave ripples in hippocampal slice preparations, indicating a crucial role of MCU in generating fast cortical network rhythms [15]. Furthermore, in cardiomyocytes, MCU regulates mitochondrial metabolism via Ca^2+^ entry, leading to increased oxidative phosphorylation (OXPHOS) and enhancing the production of reducing equivalents for ATP synthesis [16].

During action potential firing, mitochondrial calcium uptake via MCU facilitated neuronal excitability, suggesting a positive feedforward mechanism of the MCU on neuronal excitability [17]. However, the role of neuronal MCU in hyperexcitability has not been characterized yet. Due to the first evidence, MCU might serve as an attractive target from an antiepileptic perspective by inhibiting acute Ca^2+^ overload and changes in mitochondrial function. In this situation, a selective targeting of neurons, key players in hyperexcitability, is warranted. Non-selective inhibition of all brain cells, particularly of astrocytes, is likely to lead to calcium imbalance and metabolic dysfunction since astrocytes are instrumental for neurons via metabolic support [18, 19].

In this study, we aim to characterize the effect of MCU on neuronal hyperexcitability in vivo and the glioneuronal function in vitro, focusing on calcium levels, ROS, mitochondria, and metabolic adaptations.

## Materials and Methods

### Mice

Animal housing and all experimental procedures were approved by local governmental authorities (Landesamt für Natur, Umwelt und Verbraucherschutz, NRW, Germany, AZ 84–02.04.2016.A307). Mice were kept under IVC (individually ventilated cages) animal housing conditions. Mice from the Neuron (NEX)-specific Cre-driver line [20] and a loxP flanking for MCU mice (Jackson Labs (line: 029817)) were intercrossed to generate neuron-specific deletion of the MCU gene (MCUfloxNEXCre, further referred as MCU^−/−ΔN^). All littermates were proved to have shown the deletion of MCU by genotyping (Supplement Fig. 1A). These lines were on a 100% C57BL/6 background.

### In Vivo Electrophysiological Recordings

To analyze cortical network activity of mice in vivo, we recorded the spontaneous extracellular unit activity in WT and MCU^−/−ΔN^ mice. For the procedure, mice were under deep pentobarbital anesthesia (50 mg/kg i.p.), supplemented by subcutaneous injection of carprofen (Rimadyl; 5 mg/kg). Electrodes were implanted in the left hemisphere of mice targeting the hippocampal CA1 region, following stereotaxic coordinates [21]: CA1: anteroposterior − 2.00 mm, lateral 1.5 mm from bregma, and dorsoventral 1.5 mm from the brain surface (David Kopf Instruments, USA). After 7–10 days after surgical recovery, a recording of the unit activity was performed. At the end of the experiments, animals were killed by an overdose of pentobarbital (100 mg/kg, i.p), location of the electrode sites was marked by small electrolytic lesions (2.5 mA anodal current for 2 s), and brains were rapidly removed and fixed in 4% phosphate-buffered formaldehyde, pH 7.4. After completion of all experiments, the positions of the recording electrodes were histologically verified using hematoxylin and eosin staining (Supplement Fig. 1B) [22, 23]. Neuronal activity was recorded with a Multichannel Amplifier System (Alpha Omega, Israel), and unit activities were bandpass filtered at 9 kHz at a sampling rate of 40 kHz. Spikes of individual neurons were sorted by time–amplitude window discrimination and principal component analysis (Offline Sorter, Plexon Inc., Dallas, TX, USA) and verified through quantification of cluster separation, as described before [23, 24].

### Isolation of Hippocampal Glioneuronal Cultures

Neuronal cell cultures were obtained from WT and MCU^−/−ΔN^ pups (P0-1) as described before [25, 26]. In brief, brains were dissected, and the hippocampus was preparated. After mechanical and chemical dissociation, cells were seeded at a density of 150,000 cells per Coverslip (18 mm diameter). Glioneuronal cultures were incubated at 37.0 °C and 5% CO_2_ and held in culture for up to 18 days before experiments. Experiments were performed in glioneuronal cultures > 12 DIV to allow for maturation of synapses.

### Live Cell Imaging and Staining Procedures

Live cell images were acquired on an epifluorescence inverted microscope equipped with a 40 × oil immersion fluorite objective. Dyes were diluted in artificial cerebrospinal fluid (aCSF; 120 mM NaCl, 2.5 mM KCl, 1.25 mM NaH_2_PO_4_, 22 mM NaHCO_3_, 25 mM glucose, 2 mM CaCl_2_, 2 mM MgSO_4_). Live cell imaging setup provided excitation light by an LED lamp passing through a monochromator at 340, 380, or 530 nm (Carn research, Faversham, UK). Emitted fluorescence was reflected with a long-pass filter to a cooled CCD camera (Retiga; QImaging) and digitalized to 12-bit resolution.

To analyze total intracellular Ca^2+^ levels, a high-affinity radiometric fluorescent dye, fura-2-acetoxymethyl ester (Fura-2-AM), was used, and Ca^2+^ levels were detected via fluorescence microscopy [27]. Therefore, glioneuronal cultures were incubated with Fura-2-AM and 0.005% Pluronic. Cells were washed with aCSF prior to the experimental procedure. Ca^2+^ measurements with Fura-2-AM were performed in glioneuronal cultures using excitation light provided by an LED lamp, the beam passing through a monochromator at 340 nm and 380 nm. Emitted fluorescent light was reflected through a 515-nm-long-pass filter to a cooled CCD camera and digitized to 12-bit resolution. Traces are computed as ratios of excitation fluorescence at 340 and 380 nm, with emission at > 515 nm.

Intracellular ROS levels were measured using the cell-permeant reagent Dihydroethidium (DHE). Images were captured at an interval of 5 s while the cells were excited at 530 nm and stained with 16 µM dihydroethidium (DHE, D11347, Invitrogen). Cells were imaged instantly without preincubation to avoid an accumulation of oxidized products, and DHE was present for the entire recording period. 

To analyze glutathione levels, monochlorobimane (MCB) was used, which is a non-fluorescent bimane that is spontaneously permeable across cell membrane and forms a fluorescent adduct when combined with reduced glutathione (GSH) in a reaction catalyzed by glutathione s-transferase. Glioneuronal cells were stained with 50 µM monochlorobimane (MCB, M1381MP, Invitrogen) for 40 min. Cells were excited by illumination at 380 nm, and emitted light was detected at 510/80 nm.

A cell death assay was performed to analyze neurotoxicity in glioneuronal cultures. Cells were stained with propidium iodide (PI) (10 µM), which stains only dead cells, in combination with Hoechst 33342 (4.5 µM), which stains all nuclei, and a percentage of PI-positive cells in correlation to all nuclei was calculated.

To further characterize the potential changes in mitochondrial function, we used tetramethylrhodamine (TMRM) to analyze the mitochondrial membrane potential (Δψm). Cells were stained with 30 nM TMRM (T668, Invitrogen) for 40 min. TMRM was not washed out and present during the whole imaging. Cells were imaged with excitation at 530 nm and emission at 705/72 nm [26].

All experiments were performed at room temperature, and all imaging parameters were kept constant between experiments.

### Evaluation of Metabolic Function

To characterize basal metabolic changes occurring under MCU deficiency in glioneuronal cultures, the Seahorse XFe96 Analyzer (Agilent Technologies) was used. Measurements were performed according to the manufacturer’s instructions. In brief, cells were seeded at 15,000 cells per well on Seahorse XF24 tissue culture plates (Seahorse Bioscience Europe) in neurobasal media for 12 to 18 days at 37 °C and 5% CO_2_. Before the measurement, cells were washed, and neurobasal media was replaced with aCSF supplemented with 10 mM sodium pyruvate. At any condition, three consecutive measurements of OCR/ECAR were made. OCR/ECAR was evaluated under basal conditions and sequentially exposed to mitochondrial stressors (1 μM oligomycin, 0.5 μM FCCP, and 0.5 µM rotenone plus 0.5 μM antimycin A (all Merck)) [28]. For data analysis, Wave Software (Agilent Technologies) was used.

### In Vitro Model to Investigate Seizure Activity and Excitotoxicity

To simulate a pathological environment, cells were challenged with a low-Mg^2+^ extracellular medium to induce epileptiform activity in neurons [29–31]. Removing magnesium from culture medium promotes NMDA receptor activation by vesicular glutamate release, resulting in seizure-like activity and Ca^2+^ oscillations in neurons [26, 32]. Additionally, acute glutamate (100 µM) application was tested to evaluate the effect of MCU on potential excitotoxicity-induced Ca^2+^ overload.

### Statistical Analyses

Data represent mean ± SEM of at least three independent experiments. Prior to statistical analysis, all data were tested for normal distribution and detection of outliers. Data were statistically processed using a two-tailed Student’s *t*-test with two group comparisons. Comparisons between multiple groups were tested by one-way analysis of variance (ANOVA) followed by Tukey’s post hoc. *P* < 0.05 was considered statistically significant. Statistical analyses were performed using GraphPad Prism 6 (Version 6.01; San Diego, CA). Analysis for live cell Imaging raw data was done by Metafluor Fluorescence Ratio Imaging Software (Molecular Devices, LLC, Canada/USA), followed by plotting the data with Origin (V2019, OriginLab Corporation, Northampton, MA, USA) and ImageJ (V1.46r, Wayne Rasband, National Institute of Health, USA). Analyses were performed on single cells (Ca^2+^, DHE, MCB, and TMRM measurements) or regions (cell death analysis).

## Results

### MCU Deficiency Results in Less Excitability In Vivo

**Fig. 1:**
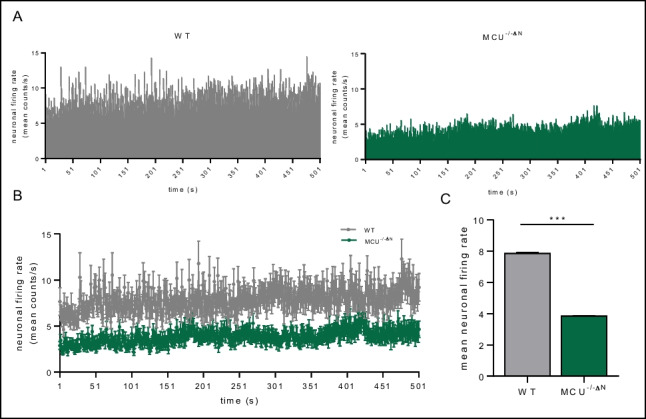
Neuronal excitability in vivo. Neuronal MCU deficiency reduces neuronal firing rate in vivo. **A** Histogram of the mean neuronal firing rate of WT (left) and MCU^−/−∆N^ (right) mice over time. **B** Comparison of neuronal firing rate over time. Bargraphs show the mean ± SEM over the complete measurement period. **C** Quantitative summary of mean neuronal firing rate over time. *n* = 8 mice/group, ***p < 0.001

Neuronal excitation is a hallmark of neuronal network function. To examine the effects of neuronal MCU deficiency, we implanted electrodes within the CA1 region of WT and MCU^−/−ΔN^ mice, and in vivo electrophysiological recordings and single unit analysis were performed to analyze the spontaneous excitability. In WT mice, this analysis revealed a consistent neuronal firing rate under resting conditions, which was constant during the observation timeframe, indicating a stable cortical network over time (Fig. 1A). In contrast, neuronal firing and excitation were significantly reduced in MCU^−/−ΔN^ animals, indicating a reduction of neuronal excitation (Fig. 1A–C).

### Intracellular Calcium Handling in MCU-Deficient Glioneuronal Cultures

**Fig. 2:**
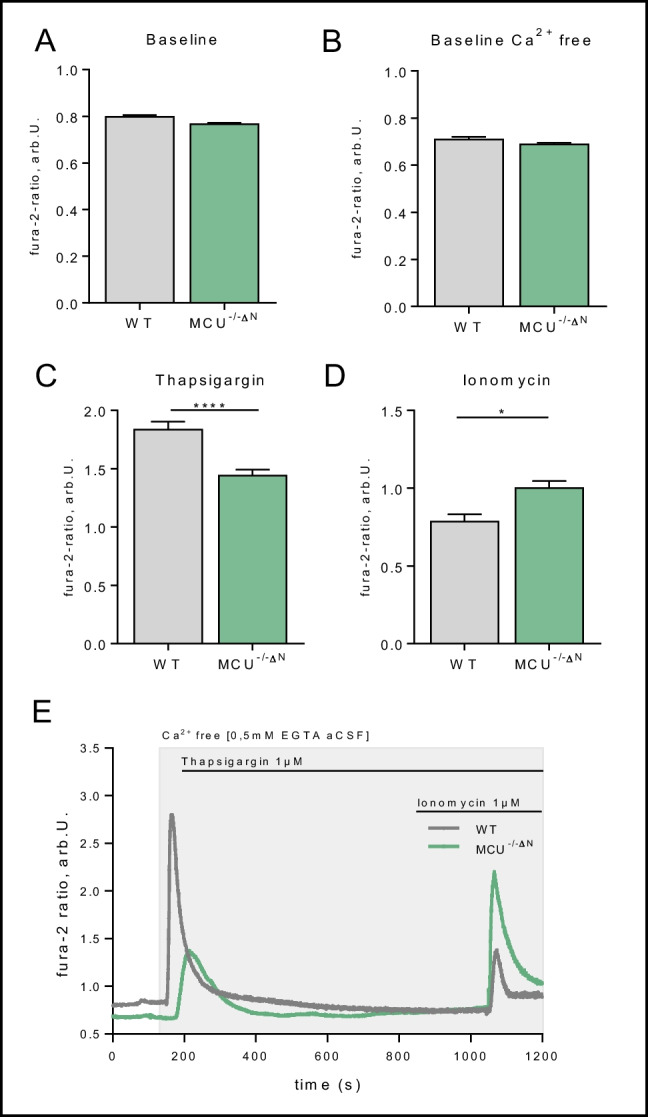
Calcium homeostasis in MCU^−/−∆N^ glioneuronal cells. **A**, **B** Baseline levels (**A**) of intracellular calcium (fluorescence ratio of 340/380 nm of Fura 2AM) in regular (*n* = 160 (WT)–204 (MCU) cells) and Ca^2+^ free media (**B**) (*n* = 185 (WT)–118 (MCU) cells). **C**, **D** Changes of calcium release in WT and MCU neurons after the addition of thapsigargin (**C**) (1 µM, *n* = 293 (WT)–315 (MCU) cells) or ionomycin (**D**) (1 µM, *n* = 185 (WT)–118 (MCU) cells). **E** Representative traces show thapsigargin and ionomycin’s effect on intracellular calcium levels in WT and MCU^−/−∆N^ neurons. All data presented as mean ± SEM, *p < 0.01, ***p < 0.001

Since MCU is responsible for intracellular Ca^2+^ transport through the inner mitochondrial membrane, we first examine intracellular calcium levels in our glioneuronal cultures. When comparing total Ca^2+^ levels under baseline condition, no difference was observed between WT and MCU^−/−ΔN^ cells, independent of extracellular calcium concentration (Fig. 2A, B). However, since MCU is known to be active in the context of stress response [33, 34], we further challenged the calcium flux by either stimulation of Ca^2+^ release of the endoplasmic reticulum (ER) using thapsigargin or mitochondrial calcium release using ionomycin. Here, we observed a decrease in calcium release from the ER with a compensatory increase in calcium release from mitochondria in MCU^−/−ΔN^ neurons compared to WT controls (Fig. 2C–E). This is rather surprising since MCU^−/−ΔN^ cells should intuitively have reduced mitochondrial Ca^2+^ release capacity because of missing mitochondrial influx through MCU. These effects lead to the hypothesis that MCU^−/−ΔN^ cells might compensate for their mitochondrial Ca^2+^ flux deficiency under baseline conditions by channeling Ca^2+^ from the ER, explaining reduced Ca^2+^ efflux by stimulation with thapsigargin. Whereas additional stimulation of mitochondrial Ca^2+^ flux is increased, potentially through augmented intramitochondrial Ca^2+^ levels due to missing release through MCU. This data indicates that cells compensate for the MCU deficiency by using other Ca^2+^ storages to balance intracellular Ca^2+^ homeostasis.

### MCU Deficiency Is Neuroprotective and Displays Reduced ROS Production

**Fig. 3:**
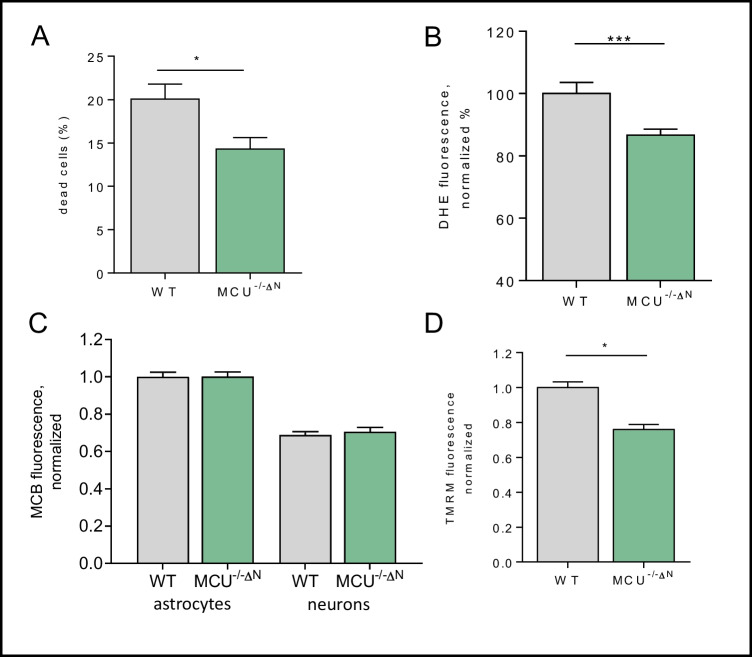
MCU deficiency in cell death and ROS homeostasis. **A** Analysis of co-staining of glioneuronal cells with propidium iodide and Hoechst 33342 to evaluate cell death (*n* = 15 (WT)–19 (MCU) regions). **B** Intracellular ROS levels are measured using DHE (*n* = 99 (WT)–149 (MCU) cells). **C** Glutathione (GSH) was measured using the fluorescent agent monochlorobimane (MCB) and analyzed in astrocytes (*n* = 31 (WT)–33 (MCU) cells) and neurons (*n* = 68 (WT)–63 (MCU) cells). **D** Measurements of the membrane potential were assessed using tetramethylrhodamine-methylester (TMRM) (*n* = 79 (WT)–91 (MCU) cells). Data are presented as foldchange to control (WT) condition. All data presented in mean ± SEM, *p < 0.01

Since we could observe that MCU^−/−ΔN^ cells seem to be less excitable and showed a shift in the intracellular calcium channeling, we aim to clarify the possible effects on cellular physiology. To evaluate the effect of MCU on cell viability, we analyzed cell death rate using propidium iodide in combination with Hoechst 33342 and observed reduced cell death in MCU^−/−ΔN^ glioneuronal cultures (Fig. 3A). Reactive oxygen species (ROS) are known to be involved in signaling, but exacerbated ROS levels are associated with cellular dysfunction and neuronal death [35]. In line with the reduction in cellular death, MCU^−/−ΔN^ cells also display a decrease in intracellular ROS levels (Fig. 3B), as shown before in other cell types [14, 36]. In addition, we further questioned the potential reasons for reduced ROS levels and, therefore, examined the major antioxidative system of glutathione (GSH) using MCB live cell imaging. As demonstrated before, we can observe that GSH levels in astrocytes are elevated in comparison to neuronal cells [37] in both WT and MCU^−/−ΔN^ cultures. However, levels of intracellular GSH are unchanged between WT and MCU^−/−ΔN^ cells (Fig. 3C), indicating other potential causes for reduced ROS levels.

### MCU^−/−ΔN^ Cells Show Reduced Metabolic Fluxes

**Fig. 4:**
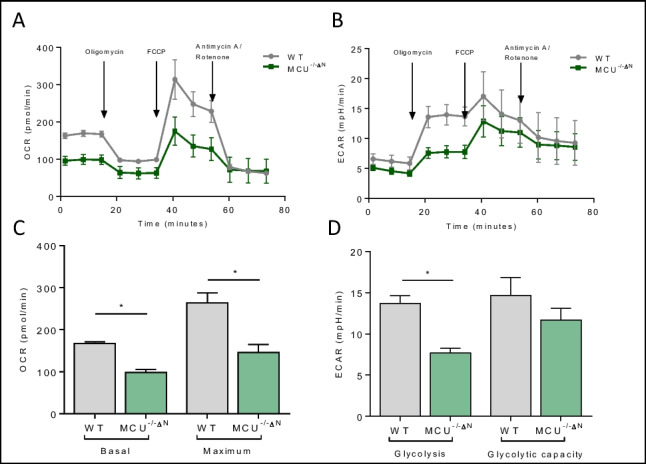
Metabolic adaptation MCU^−/−∆N^ glioneuronal cells. **A**–**D** Oxygen consumption rate (OCR) as an indicator of oxidative respiration (**A**, **C**) and extracellular acidification rates (ECAR) as indicators of glycolytic activity (**B**, **D**) of WT and MCU ^−/−∆N^ glioneuronal culture measured with the Seahorse extracellular flux analyzer. Oligomycin blocks the mitochondrial ATP synthase; FCCP is a chemical uncoupler of electron transport and oxidative phosphorylation; rotenone and antimycin A are complex I and III inhibitors (*n* = 3). **A**, **B** Graph shows OCR/ECAR over time in glioneuronal cells. All data presented in mean ± SEM, *p < 0.01

Since we observed a reduction in spontaneous excitation, we ask if MCU-deficient cells show a difference in basal mitochondrial membrane potential (MMP) since a stable MMP is needed for proper neuronal activity [38]. In line with reduced excitation in vivo, we also could observe a depolarization of MMP in MCU^−/−ΔN^ cells in the glioneuronal cultures (Fig. 3D). A stable MMP is also needed for a proper working energy metabolism involving the respiratory chain complex. However, the respirator chain complex is also a major producer of ROS. Therefore, we ask if observed decrease in ROS levels, without alteration of the antioxidative system (GSH), might be explained by alteration of metabolic energy status of MCU^−/−ΔN^ cells. To analyze metabolic function, we used the Seahorse flux analyzer. We observed that MCU^−/−ΔN^ cells show a reduction in the basal oxygen consumption rate (OCR) and maximal OCR (Fig. 4A, C), indicating an overall reduction in oxidative metabolism in these cells. Furthermore, even when neurons are known to preferentially use oxidative phosphorylation (OXPHOS) for energy supply, they also can use, at least in part, glycolysis-derived pyruvate to fuel mitochondria [39, 40]. In line with the reduction of excitation in vivo, we also observed a reduction in the glycolytic flux in MCU^−/−ΔN^ cells (Fig. 4B, D). Moreover, we used the ionophore FCCP to increase the proton leak rate to provoke a consecutive ATP demand, allowing us to determine whether the maximum glycolytic rate has been reached. However, after stimulation with FCCP, MCU^−/−ΔN^ cells did not show differences in the maximum glycolytic capacity. Overall, we can conclude that MCU^−/−ΔN^ cells display a decrease in metabolic fluxes, potentially leading to a reduction of ROS production through oxidative metabolism.

### MCU Deficiency Is Protective in Stress Response In Vitro

**Fig. 5:**
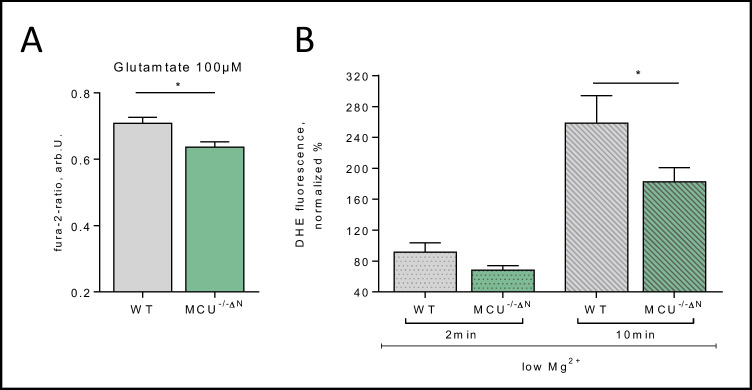
Stress response in MCU^−/−∆N^ cells. **A** Measurements of intercellular calcium in single cells using Fura 2AM after glutamate (100 µM) exposure in WT and MCU^−/−ΔN^ neurons (*n* = 150 (WT) vs. 157(MCU) cells). **B** Intracellular ROS levels measured with DHE. To simulate stress conditions during hyperexcitability, the medium was changed to a low Mg^2+^ medium, and ROS increase was measured after 2 min and 10 min (*n* = 99 (WT)–149 (MCU) cells). All data presented in mean ± SEM, *p < 0.01

Since it is known that MCU activity is stress-related, we further examine if MCU^−/−ΔN^ cells show a different response to external stressors. We, therefore, first used the glutamate excitotoxicity model, and indeed, the excessive Ca^2+^ influx seen in WT cells was reduced in MCU^−/−ΔN^ cultures, indicating a potential protection for Ca^2+^ overload in MCU^−/−ΔN^ cells (Fig. 5A).

To further evaluate the effects of MCU^−/−ΔN^ as a potential therapeutic target in epilepsy, we additionally used an in vitro hyperexcitability model (low Mg^2+^ model) to test our hypothesis of potential protective MCU deficiency due to reduced ROS levels. Indeed, as seen under baseline conditions, MCU^−/−ΔN^ cells also show reduced ROS levels under low Mg^2+^ conditions, indicating MCU as potentially protective in situations of excessive ROS production (Fig. 5B).

## Discussion

Since the initial discovery of the MCU channel in 2004, knowledge about its function arose quickly. MCU is involved in stress/pathological reactions in various cell types and is supposed to regulate the activation of a cell death cascade. In neurons, MCU overexpression increased mitochondrial Ca^2+^ levels following NMDA receptor activation, resulting in excessive Ca^2+^ influx, which results in MMP depolarization leading to neuronal death [9]. Therefore, manipulating MCU is an attractive target to influence intracellular Ca^2+^ fluxes and thereby reduce potential Ca^2+^ overload, as seen in seizures and hyperexcitability [26]. In line with our observation of reduced MMP and reduction in glutamate-dependent Ca^2+^ influx under MCU deficiency, it was shown before that pharmacological inhibition (using Ru360) of MCU can prevent the glutamate-dependent Ca^2+^ influx [41], a glutamate-dependent increase of MMP (depolarization) [42] and glutamate-induced mitochondrial swelling [43]. However, more interesting, we observed that MCU deficiency results in a different channeling of intracellular Ca^2+^ storages since stimulation with thapsigargin (stimulation of ER Ca^2+^ release) reduces Ca^2+^ fluxes. In contrast, additional stimulation of mitochondrial Ca^2+^ release (ionomycin) is increased in MCU-deficient cells, indicating a potential compensatory mechanism of calcium handling in MCU-deficient cells. This observation might explain, at least in part, the effects seen by others, whereas inhibition of MCU only partially inhibits Ca^2+^ flux in the matrix of isolated mitochondria [11, 44]. In contrast, deletion of MCU completely inhibited Ca^2+^ uptake in the liver, heart, and skeletal muscle mitochondria [11], indicating a cell type-dependent function of MCU.

Here, we demonstrate that neuronal MCU deficiency reduces cellular death, which might be an effect of reduced ROS production. In line with our results, it was shown before that the pharmacological inhibition of MCU leads to improved mitochondrial morphology and functional stability [45], which results in reduced cell death in the ischemia/reperfusion model [34]. In addition, pharmacological MCU inhibtion was shown to reduce, but not completely prevent, the opening of the mitochondrial permeability transition pore (PTP), which might additionally explains at least in part reduced cell death in MCU-deficient cells [11, 46]. In contrast, activation of MCU with kaempferol leads to an increase in ROS levels [33], and MCU overexpression in vivo is sufficient to trigger gliosis and neuronal loss [47], highlighting the potential of MCU inhibition from a neuroprotective perspective. Notably, all effects in our study were shown in a model of neuron-specific MCU deficiency in glioneuronal cultures. These findings indicate that the protective effect is not hampered by astrocytic MCU function. Furthermore, we deliberately chose this approach as global antagonism of MCU is likely no therapeutic strategy due to the ubiquitous expression of MCU in multiple organs.

In our study, we can demonstrate that neuronal MCU deficiency leads to reduced excitation in vivo and reduces ROS levels in a hyperexcitability model in vitro. It is known that excessive intramitochondrial Ca^2+^ levels can lead to depolarization of MMP [33]. Therefore, it is counterintuitive that MCU deficiency leads to a reduction of MMP. However, in a physiological setting, mitochondria increase oxidative phosphorylation during periods of stress to meet increased metabolic demand. However, under pathological condition of hyperexcitation, this stress response can be exhausted, resulting in neuronal death. In line with previous results [48], we could demonstrate that MCU-deficient cells showed a decrease in oxidative phosphorylation, which might be explained by the reduction in ROS levels and MMP. This metabolic alteration potentially prevents cells from excess stress reaction by counteracting stress response by rebalancing the ROS homeostasis through metabolic pathway modifications. Oxidative stress is known to occur in pathogenesis of acquired epilepsies [49], but is also involved in pathogenesis of psychiatric disorders, such as depression [50, 51], indicating MCU as a potential target in bidirectional therapeutic option in epilepsy and their comorbidities.

In addition, we also observed a decrease in glycolytic flux measured by the ECAR flux analyzer in neuronal MCU-deficient glioneuronal cultures. It is known that neurons mainly rely on oxidative metabolism to sustain their energy metabolism. However, the glycolytic pathway’s metabolites are needed to maintain mitochondrial function [40]. In comparison to astrocytes, glycolytic rates in neurons are relatively low. Using our neuron-specific approach, we can reveal that the glycolytic distress seen in our glioneuronal cultures under MCU deficiency affects glycolytic downstream pathways in neurons. To correctly interpret the ECAR data, it has to be taken into account that measurements of ECAR rely on consumption that the generated proton efflux (H^+^) is equivalent to the glycolytic flux within cells [52]. By blockade of the respiratory chain using oligomycin, we can conclude that the measured proton efflux is a result of the cellular glycolytic flux. However, it is known that neurons become energy/metabolite supplied by astrocytes via lactate [39], which might be reused for fuelling the TCA cycle via pyruvate, leading to an additional increase in proton levels [53]. Therefore, we conclude that MCU deficiency might affect neuronal glycolysis but might also influence mitochondrial function via the TCA cycle. However, the underlying mechanisms of these effects are still elusive.

It is known that MCU gets activated through larger intracellular Ca^2+^ levels as seen under pathological conditions [54], and increased Ca^2+^ levels are involved in hyperexcitation and trigger opening of the membrane permeability transition pore (PTP). Under pathological conditions such as epileptic seizures and excitotoxicity, this increased Ca^2+^ entry via the MCU might affect the tightly regulated mitochondrial energy metabolism. Novorolsky et al. demonstrated that acute siRNA-mediated MCU knockdown protected cortical neuron cultures from mitochondrial deficits by resembling mitochondrial respiration following oxygen and glucose deprivation [55], which might prevent cells from metabolic exhaustion in pathological conditions.

In conclusion, we could demonstrate that neuronal MCU deficiency results in a neuroprotective phenotype under stress conditions as hyperexcitation. This might be the effect of reduced oxidative stress, potentially modulated by different Ca^2+^ channeling and neuronal metabolic adaptations. These results indicate MCU as a potential target in future neuroprotective therapies.

## Supplementary Information

Below is the link to the electronic supplementary material.

Supplementary file1 (DOCX 466 KB)

## Data Availability

No datasets were generated or analysed during the current study.
