# Supplementary material for: Neuronal Mitochondrial Calcium Uniporter (MCU) Deficiency Is Neuroprotective in Hyperexcitability by Modulation of Metabolic Pathways and ROS Balance
Source: Mol Neurobiol. 2024 Apr 23;61(11):9529–38. doi: 10.1007/s12035-024-04148-x (PMC11496325; doi:10.1007/s12035-024-04148-x)
Supplement: Supplementary file 1 — Supplementary file1 (DOCX 466 KB) [file 12035_2024_4148_MOESM1_ESM.docx]

## Supplementary Figures


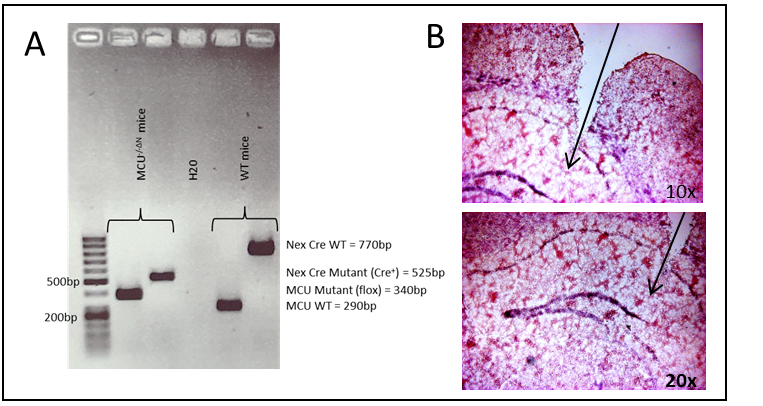


**Supplement Figure 1**

**A** Representative genotyping of MCU^-/-ΔN^ mice showing MCU mutant and NexCre expression. **B** H&E stainings of mouse brain showing the hippocampal structures. Representative Image of electrode position (arrows) during in vivo recordings.
